# Retrospective Side Effect Profiling of the Metastatic Melanoma Combination Therapy Ipilimumab-Nivolumab Using Adverse Event Data

**DOI:** 10.3390/diagnostics8040076

**Published:** 2018-10-31

**Authors:** Theodoros G. Soldatos, Antonia Dimitrakopoulou-Strauss, Lionel Larribere, Jessica C. Hassel, Christos Sachpekidis

**Affiliations:** 1 Molecular Health GmbH, 69115 Heidelberg, Germany; 2 Clinical Cooperation Unit Nuclear Medicine, German Cancer Research Center, 69120 Heidelberg, Germany; a.dimitrakopoulou-strauss@dkfz.de (A.D.-S.); christos_saxpe@yahoo.gr (C.S.); 3 Skin Cancer Unit, German Cancer Research Center (DKFZ), 69120 Heidelberg, Germany; l.larribere@dkfz.de; 4 Department of Dermatology, National Center for Tumor Diseases, University Hospital Heidelberg, 69120 Heidelberg, Germany; Jessica.Hassel@med.uni-heidelberg.de

**Keywords:** side effects, ipilimumab, nivolumab, melanoma, real world data, data mining, pharmacoepidemiology, proportional reporting ratio

## Abstract

Recent studies suggest that combining nivolumab with ipilimumab is a more effective treatment for melanoma patients, compared to using ipilimumab or nivolumab alone. However, treatment with these immunotherapeutic agents is frequently associated with increased risk of toxicity, and (auto-) immune-related adverse events. The precise pathophysiologic mechanisms of these events are not yet clear, and evidence from clinical trials and translational studies remains limited. Our retrospective analysis of ~7700 metastatic melanoma patients treated with ipilimumab and/or nivolumab from the FDA Adverse Event Reporting System (FAERS) demonstrates that the identified immune-related reactions are specific to ipilimumab and/or nivolumab, and that when the two agents are administered together, their safety profile combines reactions from each drug alone. While more prospective studies are needed to characterize the safety of ipilimumab and nivolumab, the present work constitutes perhaps the first effort to examine the safety of these drugs and their combination based on computational evidence from real world post marketing data.

## 1. Introduction

The introduction of immune-checkpoint inhibitors in recent years constitutes a major breakthrough in the treatment of advanced melanoma. With this approach, specific immune checkpoints are blocked, such as the cytotoxic T lymphocyte antigen-4 (CTLA-4) and the programmed cell death protein-1 (PD-1), enhancing thus antitumor immunity. Three checkpoint inhibitors have been associated with objective responses in metastasized melanoma and have been approved by the US Food and Drug Administration (FDA): ipilimumab, nivolumab and pembrolizumab [1,2,3,4,5,6]. Ipilimumab targets CTLA-4, while nivolumab and pembrolizumab target PD-1 (Supplementary Material S1, Figure S1).

Although these single-agent treatment strategies have a proven efficacy in advanced melanoma, not all patients benefit from them. Moreover, tumor control on a long-term basis remains a big challenge of immunotherapy. In this context, combinational treatment represents a reasonable approach for enhancing antitumor activity. In particular, the combination of concurrent PD-1 and CTLA-4 blockade holds considerable promise, since PD-1 contributes to T-cell exhaustion primarily in peripheral tissues and within the tumor microenvironment, and CTLA-4 inhibits at earlier point in T-cell activation [7,8]. In other words, PD-1 and CTLA-4 blockade act in distinct positions within the T-cell response pathway and complement, rather than overlap one another. Nivolumab with ipilimumab has been the first combination treatment to demonstrate a long-term benefit in advanced untreated melanoma: in a phase III trial (CheckMate 067) involving 945 patients with previously untreated, unresectable stage III or IV melanoma, the median progression free survival for the combination treatment nivolumab plus ipilimumab was 11.5 months, as compared to 2.9 months with ipilimumab and 6.9 months with nivolumab [9]. Recently, an updated analysis of the trial was published according to which the overall survival (OS) rate at three years was 58% in the nivolumab plus ipilimumab group, 52% in the nivolumab group, and 34% in the ipilimumab group [10]. These results indicate that nivolumab combined with ipilimumab is superior to ipilimumab and likely also nivolumab alone.

Yet, the increased immune activity triggered by immunotherapeutic agents is associated with inflammatory side-effects, commonly addressed as immune-related adverse events (irAEs). The precise pathophysiologic mechanism of these, sometimes severe, adverse events (AEs) is not yet clear and no prospective studies on their successful management have been published. Although potentially all organs may be affected, irAEs usually occur in the gastrointestinal tract, endocrine glands, skin and liver, while the pulmonary, musculoskeletal, and central nervous systems are less often involved [11]. Reasonably, more concerns regarding toxic effects rise when two immunotherapeutic agents are combined. Specifically, 55% of the CheckMate 067 trial’s patients demonstrated grade 3 or 4 AEs when nivolumab and ipilimumab were used together, in comparison to 16.3% and 27.3% of the nivolumab and ipilimumab monotherapy groups, respectively [9]. The safety profile was similar also in the updated survival analysis of the trial with grade 3 or 4 treatment-related AEs occurring in 59% of the patients in the nivolumab plus ipilimumab group, in 21% of those in the nivolumab group, and in 28% of those in the ipilimumab group [10].

## 2. Materials and Methods

To examine the side-effect profiles of ipilimumab, nivolumab, and their combination in metastatic melanoma patients, we analyzed serious AEs from the FDA’s Adverse Event Reporting System (FAERS).

### 2.1. The Adverse Event Data Set

We performed our analysis over the publicly available FAERS data set (incl. 2017Q2) that contained 9.5 million reports for 7.9 million cases. FAERS information used in this analysis included patients’ treatments (medications), indications (disease or condition), as well as reported adverse reactions and observed outcomes (e.g., “death” or “hospitalization”). 

### 2.2. FAERS Data Integration

FAERS data regarding treatments, indications, and adverse reactions were consolidated in the following way:

- FAERS medication synonyms (coded in free-text names) were mapped to drugs and compounds from DrugBank [12] and PubChem [13].
- Drugs were further categorized according to the Anatomical Therapeutic Chemical (ATC) classification system.
- Indications and reactions (coded by FAERS in terms from the MedDRA dictionary) were contextualized further by using the full hierarchical structure of the ontology.

### 2.3. Statistical Characterization of the Adverse Event Data

For the statistical characterization of the observed associations between the reported in FAERS drugs, reactions, and outcomes, we employed the proportional reporting ratio (PRR), an established metric of disproportionality in pharmacovigilance. PRR gives an indication for the relative congruence of pairwise entity relations as based on their co-occurrence in subsets of AE data [14]. For a drug (D) and an event (E) the PRR metric is defined to be the value of *a(c+d)/c(a+b)*, based on the following contingency matrix (1).

AE cases
**Event (E)**

**Not E**
Totals
**Drug (D)**

*a*

*B*

*a + b*

**Not D**

*c*

*D*

*c + d*
Totals
*a + c*

*b + d*

*N = a + b + c + d*


An event E may represent the occurrence of AE reaction(s) or of patient outcome(s). While PRR has become a standard metric for the evaluation of safety signals, we also considered relevant statistical significance be reflected by Fisher’s exact test *p*-values (two-tailed).

### 2.4. Experiments: Definition of Cohorts

**Figure 1:**
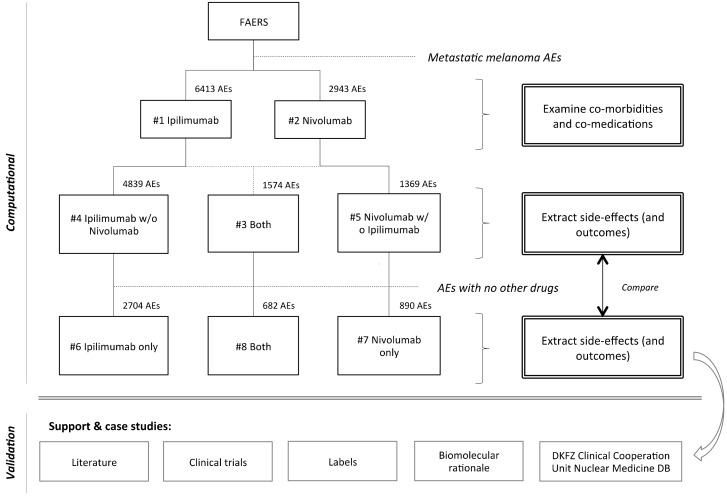
Overview of the side effect profiling approach. Metastatic melanoma patient cohorts treated with ipilimumab and/or nivolumab were identified and a virtual trial was performed, in which we compared reaction occurrence in each set of AEs. First we profiled the reported AEs in patient cohorts receiving only ipilimumab and/or nivolumab (cohorts #6–#8). These safety profiles were similar to those of the wider cohorts, in which ipilimumab and/or nivolumab were co-administered with other drugs (cohorts #3–#5). We also reviewed literature to discuss the biological rationale underlying irAEs and present case studies regarding potential clinical implications.

To examine drug-reaction occurrences, we defined patient cohorts as collections of AE cases that contained each drug in the context of metastatic melanoma. The identified cohorts were then examined with respect to reported drugs, indications, reactions, and outcomes. Results per cohort are described in terms of observed case counts (i.e., number of AEs a certain occurrence was observed in), percentage of cohort cases, and the PRR disproportionality score. Metastatic melanoma cases were defined to be those cases for which there was an indication reported that linked under the MedDRA hierarchy to the term ‘Skin melanomas (excluding ocular)’, such as ‘MALIGNANT MELANOMA’, ‘METASTATIC MALIGNANT MELANOMA’, or ‘MALIGNANT MELANOMA STAGE IV’. Specifically, eight metastatic melanoma cohorts were defined (Figure 1):

- **#1) Ipilimumab:** cases that had ipilimumab reported as medication. This set of 6413 AEs was used to profile co-morbidities and co-medications of ipilimumab, as reported in FAERS.
- **#2) Nivolumab**: cases that had nivolumab reported as medication. This set of 2943 AEs was used to profile co-morbidities and co-medications of Nivolumab, as reported in FAERS.
- **#3) Ipilimumab and Nivolumab (together)**: cases that had both ipilimumab and nivolumab reported as medications. This set of 1574 AEs was used to cross-validate the combination’s side-effect profile when used together with other drugs.
- **#4) Ipilimumab without Nivolumab**: cases that had ipilimumab reported as medication but not nivolumab. This set of 4839 AEs was used to cross-validate the ipilimumab side-effect profile when used together with other drugs, but not with Nivolumab.
- **#5) Nivolumab without Ipilimumab:** cases that had nivolumab reported as medication but not ipilimumab. This set of 1369 AEs was used to cross-validate the nivolumab side-effect profile when used together with other drugs, but not with ipilimumab.
- **#6) Ipilimumab (only):** cases that had only ipilimumab reported as medication, and no other drugs. This set of 2704 AEs was used to identify the ipilimumab side-effects.
- **#7) Nivolumab (only):** cases that had only nivolumab reported as medication, and no other drugs. This set of 890 AEs was used to identify the nivolumab side-effects.
- **#8) Ipilimumab and Nivolumab (only)**: cases that had both ipilimumab and nivolumab reported as medications, and no other drugs. This set of 682 AEs was used to identify the combination’s side-effect profile.

To systemize the statistical characterization, each cohort’s AEs (noted as ‘D’ in contingency matrix 1) were compared against the remaining FAERS (noted as ‘Not D’) during the calculation of PRR signals and for Fisher’s exact test.

### 2.5. Analysis of Outcomes

To compare the occurrence of outcomes ‘death’ and ‘hospitalization’ in the presence and absence of selected reactions in each cohort, we employed an enrichment approach [15,16] without multiple test correction and with PRR as ‘enhancement factor’, based on the following contingency matrix (2).

Cohort’s AEs
**Outcome (O)**

**Not O**
Totals
**Reaction (R)**

*A*

*b*

*a + b*

**Not R**

*C*

*d*

*c + d*
Totals
*a + c*

*b + d*

*N = a + b + c + d*


### 2.6. Patient Case Examples

For data-supportive and illustrative reasons, the database of the Clinical Cooperation Unit Nuclear Medicine of the German Cancer Research Center (DKFZ) was searched for examples of melanoma patients undergoing immunotherapy with radiologic signs indicative of irAEs. No further analysis regarding these data was performed in the present study.

## 3. Results

**Figure 2:**
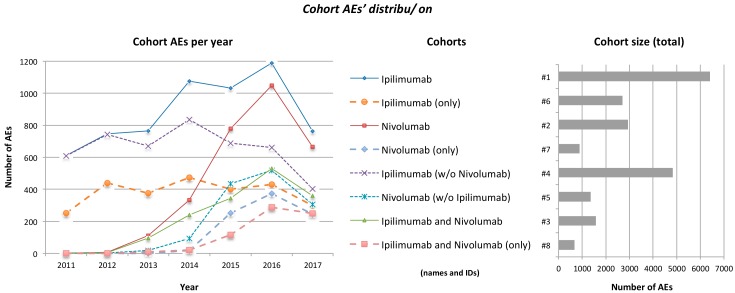
Distribution of cohorts’ AEs over time (**left**) and of cohorts’ total size (**right**). Distribution over time does not include incomplete AEs for which no date was specifically registered or AEs reported prior to 2011; AEs dated prior to a drug’s approval may reflect reports from preapproval studies and clinical trials. The reduction of AE numbers for 2017 is explained by the fact that the full dataset for that year was not yet released by FAERS at the time this analysis took place. FDA approved ipilimumab on 2011 and nivolumab on December 2014.

Before starting the analysis we wanted to verify that the identified patient cohorts reflected properly the use of ipilimumab, nivolumab, and their combination in the clinical practice of metastatic melanoma. Indeed, cohort AE timelines revealed reduction of ipilimumab monotherapy use over time, as opposed to the gradual increase of nivolumab use and of the ipilimumab plus nivolumab combination (Figure 2). We also examined co-morbidities (indications of co-medications) mentioned in cohorts #1–#5. The highest occurrence of the top most-mentioned (non-metastatic melanoma) indication was observed in cohort #3 (namely, ‘pain’ in 3.2% of ipilimumab plus nivolumab cases). The defined cohorts were specific to melanoma patients undergoing ipilimumab and/or nivolumab treatment, since mentioned co-administered medications (such as analgesics, antipyretics, corticosteroids, non-steroid anti-inflammatory dugs, antidiarrheals, anti-infective agents) did not exhibit antineoplastic activity, but were rather applied to alleviate symptoms or coexisting conditions.

### 3.1. Side Effect Profiling

Then occurrence of reactions in each cohort was examined (Supplementary Material S2). Table 1 summarizes the twenty most frequently reported reactions in the patient cohorts that received only ipilimumab, nivolumab, and their combination, and no other drugs (cohorts #6–#8). Reactions involved both general symptoms (such as ‘fever’, ‘fatigue’), and more specific organ- or system-related (dermatologic, pulmonary, hepatic, renal, gastrointestinal) conditions, sometimes with frequency >3%.

To compensate for the reporting in FAERS of different terms that may refer to similar conditions (e.g., ‘renal failure’, ‘renal failure acute’, ‘renal impairment’), we examined reaction occurrence also at High-Level Terms (HLT) of the MedDRA hierarchy. This allowed us to filter some reactions based on their signals: e.g., ‘Asthenic conditions’, the more general category of ‘Fatigue’, was found not to be statistically significant, while some other general reaction categories were found to have high PRR signals despite their small occurrence. Supplementary Material S1’s Table S1 summarizes the examined HLT categories, mentioning also most representative reactions from each class.

Using this approach we derived side-effect profiles for ipilimumab, nivolumab, and their combination:

- **#6: Ipilimumab (only)**: diarrhoea (14.35%); colitis (11.06%); rash (6.51%); hypophysitis (4.62%); pyrexia (3.96%); pruritus (2.92%); dehydration (2.89%); decreased appetite (2.74%); anaemia (1.70%); adrenal insufficiency (1.55%); sepsis (1.41%); hepatitis (1.33%); intestinal perforation (1.29%); hyponatraemia (1.29%); hypothyroidism (1.26%); pneumonitis (1.22%); renal failure acute (1.11%).
- **#7: Nivolumab (only)**: hypothyroidism (4.72%); diarrhoea (4.27%); pruritus (3.26%); decreased appetite (2.36%); colitis (2.02%); pneumonitis (1.79%); hepatic function abnormal (1.69%); leukoderma (1.69%); pituitary analyses anterior (1.35%); muscle infections and inflammations (1.35%); sepsis (1.24%); acute kidney injury (1.24%); infusion related reaction (1.12%); uveitis (1.12%); adrenal insufficiency (1.01%); liver disorder (1.01%).
- **#8: Ipilimumab and Nivolumab (only)**: diarrhoea (9.82%); colitis (9.53%); pyrexia (7.04%); rash (5.57%); hypophysitis (3.52%); pneumonitis (3.08%); hyperthyroidism (2.93%); hypothyroidism (2.93%); pruritus (2.79%); hepatitis (2.64%); pneumonia (2.64%); dehydration (2.05%) type 1 diabetes mellitus (1.61%); liver disorder (1.61%); myocarditis (1.61%); sepsis (1.61%); adrenal insufficiency (1.47%); acute kidney injury (1.47%); pituitary analyses anterior (1.32%); arthritis (1.32%); hepatic function abnormal (1.17%).

**Figure 3:**
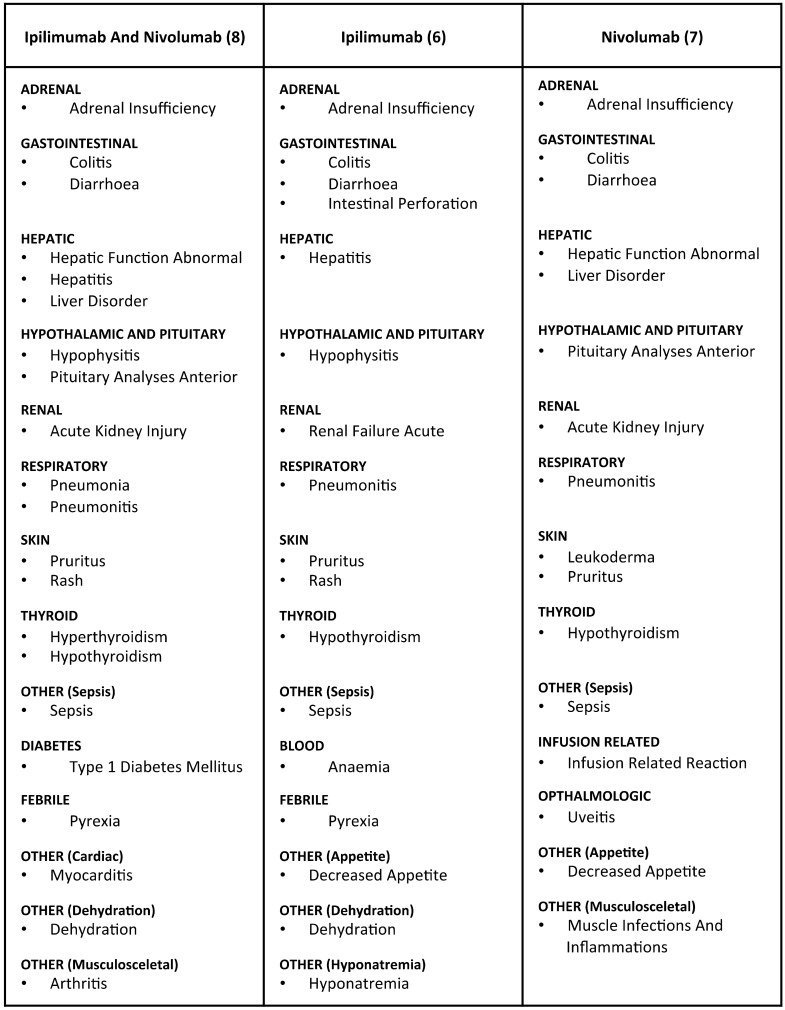

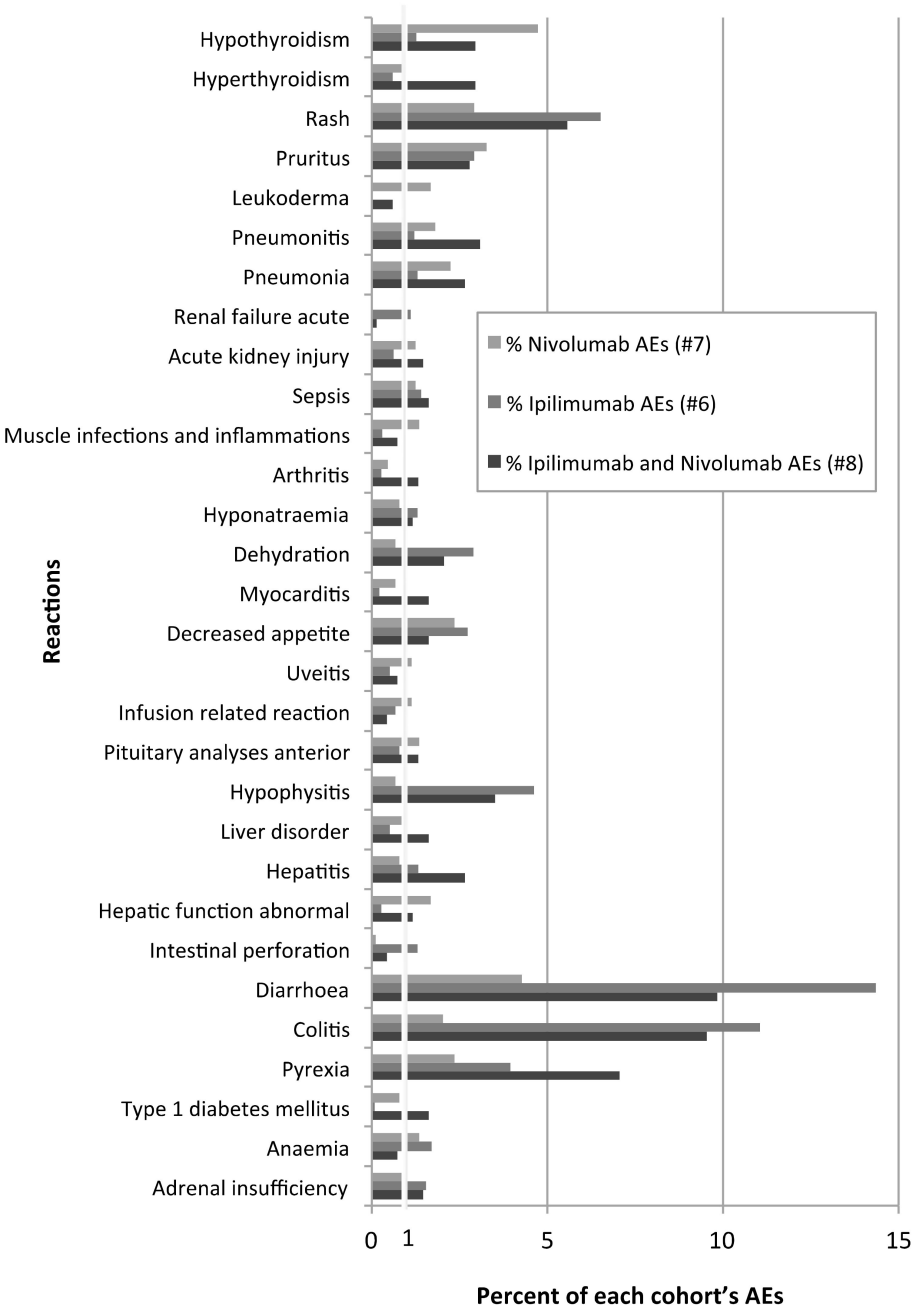
The safety profiles of ipilimumab, nivolumab and their combination. The table (**upper**) lists the identified reactions organized by affected organ/system. For the selection, statistical significance (*p*-value < 0.05), PRR signal > 1, and occurrence in at least 1% of a cohort’s AEs (**lower**) were required. While some reactions were specific to one set, many were reported in all three, but with different signals. For example, ‘Hypophysitis’ was reported in 125 AEs of the ipilimumab cohort and in 24 AEs of the ipilimumab plus nivolumab cohort—when, in comparison, overall it was mentioned in a total of 475 AEs in FAERS—thus rendering it the reaction with the highest PRR signal for these cohorts (PRR#6: 1051; PRR#8: 621). Similarly, ‘Leukoderma’ had the strongest PRR signal among nivolumab reactions (PRR#7: 2439), reported in fifteen AEs of this cohort, when there were only seventy AEs in FAERS mentioning it altogether. Similarly, ‘Myocarditis’ (with 2713 AEs in FAERS) was reported in eleven AEs of the ipilimumab plus nivolumab combination cohort (PRR#8: 47.5), and ‘Colitis’ (with 12513 AEs in FAERS) was reported in 299, eighteen, and 65 AEs of the ipilimumab, nivolumab, and the ipilimumab plus nivolumab cohorts (PRR#6: 72; PRR#7: 12.9; PRR#8: 61), respectively.

The results suggest that the identified irAEs are specific for these drugs (Figure 3). This is supported, first, by the fact that the patients of these cohorts were not co-administered any additional medications. Then, the overall occurrence of some reactions in FAERS was at times small, but very specific to the examined cohorts (e.g., ‘Hypophysitis’ or ‘Leukoderma’). Furthermore, the reaction profiles of ipilimumab, nivolumab, and their combination when co-medicated with other drugs (cohorts #3–#5; Supplementary Material S1, Table S2) were similar to those of the more specific groups (cohorts #6–#8).

Altogether, ipilimumab had more reported side-effects than nivolumab (1130 and 675 reactions in cohorts #6 and #7, respectively), whereas their combination involved effects from both profiles (591 reactions in cohort #8)—sometimes with increased PRR signal, compared to single drug administration of either nivolumab or ipilimumab (e.g., ‘hepatitis’ or ‘pneumonitis’).

### 3.2. Outcome Analysis

Next, it was investigated whether specific side-effect manifestation might potentially be associated with the outcome of patients. Results were somewhat inconclusive when reactions were examined individually in each cohort (Supplementary Material S2). However, when examined together (all the reactions of each respective side-effect profile), we found that death occurrence was reduced and, in comparison, hospitalizations were increased in all cohorts (Supplementary Material S1, Figure S2).

## 4. Discussion

In an attempt to investigate the side-effect profiles of ipilimumab, nivolumab, and their combination therapy, we examined respective reaction occurrence in AEs from public FAERS. FAERS contains valuable information for a large amount of patients (7.9 million cases) coming from healthcare professionals, consumers, and manufacturers. Our results are based on real world events and provide additional insight to previous safety profiling efforts of those drugs—and their combination—that were based on clinical trials and translational studies.

In the context of our work, public FAERS was used for hypothesis generation only. Although FAERS data and generated signals are used regularly to evaluate safety concerns by the FDA, identified relationships should be investigated further. We make available our results and provide observed data for future reference, but these should not be interpreted as calculated incidences of events based on AE data, since this is not the intended use of FAERS. Reasons for that include, firstly, the practical inability for follow-up or on-site verification of the truth of reported AE information. Moreover, the existence of a plausible causal connection between drug exposure and an AE cannot be always proven, since reports do not always contain enough detail to properly evaluate an event. Furthermore, the FDA does not receive reports for every AE. Finally, absolute and relative frequencies or risk of AEs cannot be determined from FAERS alone unless drug exposure or appropriate control group data are available. Therefore, we only report on AE occurrence and study identified signals that indicate relationships without inferring causality only by looking at these data.

Despite the above-mentioned shortcomings, one key advantage of FAERS is the ability to examine cases from significantly more patients than in other studies. Importantly, in our work we also corrected for limitations possibly introduced from confounded cases and possible miscoding.

The main contribution of the presented results is that they not only recapitulate but also add real world evidence to the existing safety profiles of these agents. Compared to label notes, clinical trial results and other studies [9,17,18], FAERS content captured occurrence of irAEs better than that of general symptoms (such as diarrhea or fatigue), indicating a possible reporting bias for the latter—perhaps not reported as often in these FAERS cohorts. Put together, we found that ipilimumab has a higher number of reported AEs than nivolumab and the combination regimen. This could be attributed to the earlier FDA approval of ipilimumab and, therefore, its longer usage, which may also explain the reporting of a broader reaction variety in FAERS. Also, the therapeutic usage of the newer agent nivolumab, or of the emerging ipilimumab plus nivolumab combination, is much more recent, therefore collecting additional post-market pharmacovigilance data may be required to follow-up our analysis. 

One other limitation of the present study is that while FAERS contains serious AEs, the severity of each reported reaction is not specifically graded. Moreover, during the analysis of outcomes we could not compensate for the lack of information regarding treatment durations or the exact disease stage of patients. Given the limitations of the dataset we could not establish specific relations between the manifestation of individual reactions and an overall improved condition, other than the general observation that reduced death occurrence was simultaneously accompanied by an increase of hospitalizations in all cohorts when the reactions of each safety profile were examined together. While an association between the occurrence of irAEs and the efficacy of the immune checkpoint blockade has been suggested before [19,20,21], a recent retrospective study on ipilimumab showed that neither OS nor time to treatment failure were affected by the occurrence of irAEs or the need for systemic corticosteroid use [22].

Interestingly, our results suggest that the co-administration of ipilimumab and nivolumab leads to a toxicological profile that combines side-effects of both agents. When compared with results from the phase 3 CheckMate 067 study [9] and its 3-year OS analysis [10], our findings confirm the high occurrence of gastrointestinal (diarrhea, colitis), skin-related (pruritus, rash) and pyrexia events. Endocrine AEs, which are particularly significant because they may require long-term hormone therapy, were also reported, with hypophysitis (most likely attributed to the impact of ipilimumab [23]) and thyroid dysfunction (frequent among patients receiving anti-PD-1 therapy [11]) being the most common.

The specificity of certain irAEs to those immunotherapeutic agents revealed by our study, translates also into potentially significant clinical implications for the management of patients treated with ipilimumab and/or nivolumab. While the biological mechanisms underlying irAEs are still to be elucidated, their potential pathophysiologic basis involves two main courses of evidence. First, checkpoint inhibitors play key role in autoimmune conditions and secondly, tumor neoantigens and antigens from healthy tissue could cross-react (Supplementary Material S1, Figure S1) [24,25]. Importantly, irAEs are very often associated with radiologic manifestations, despite sometimes being clinically silent (Supplementary Material S1, Figure S3).

Recently, a study based on pooled trial data from the European Medicines Agency of 1551 patents with advanced melanoma demonstrated that irAEs occur more frequently for the ipilimumab plus nivolumab combination, with a shorter time-to-onset, and tend to be more severe [26]. The study provides also clinical recommendations that may render such events reversible and highlights the significance of early diagnosis and of the good communication and close cooperation of an interdisciplinary network required for more successful irAE evaluation.

## 5. Conclusions

Recent evidence suggests combining ipilimumab and nivolumab for improved clinical efficacy in metastatic melanoma patients. The enhanced immune activity triggered by these agents is frequently associated with increased toxicity risk, with irAEs being the main manifestation. Our present study describes safety profiles of ipilimumab and/or nivolumab from the retrospective analysis of AEs, based on computational evidence from real world post marketing data.

Our results suggest that the co-administration of ipilimumab and nivolumab leads to a toxicological profile that combines side-effects of both agents. These findings confirm high occurrence of gastrointestinal (diarrhea, colitis), skin-related (pruritus, rash) and pyrexia events. Endocrine AEs were also reported, with hypophysitis and thyroid dysfunction being the most common. Last, in this dataset ipilimumab had a higher number of reported AEs than nivolumab and the combination regimen.

The present study also highlights that computational analysis of real world data can translate into clinical insight for more informed patient management. We therefore invite for more computational approaches that strive to efficiently support the gain of clinical insight directly from the analysis of real world data and hope that our findings will provide additional context and scope to the efforts to interpret the impact and results of many currently active or recruiting clinical trials that study the ipilimumab plus nivolumab combination.

## Figures and Tables

**Table 1:** Top twenty most frequently reported reactions in each ‘clean’ cohort (#6–#8). The table lists a total of forty reactions, most frequently reported in each cohort, after filtering for statistical significance (Fisher’s exact test *p*-value < 0.05), PRR signal > 1, and excluding terms either reflecting the patients’ disease or not describing physiological effects (namely, ‘transfusion’, ‘death’, ‘malignant neoplasm progression’, ‘neoplasm malignant’, ‘prescribed overdose’, ‘adverse event’, ‘inappropriate schedule of drug administration’). In total there were 1484 reaction terms reported in all three cohorts, indicating overlap between the mentioning of reactions in the sets: 1130, 675, and 591 reaction terms were reported in the 2704 AE of the ipilimumab (only), 890 AEs of nivolumab (only), and 682 AEs of the ipilimumab and nivolumab (only) cohorts, respectively.

Cohort	Ipilimumab and Nivolumab (#8)			Ipilimumab (#6)			Nivolumab (#7)		
Order	Name	AEs	%	Name	AEs	%	Name	AEs	%
1	Diarrhoea	67	9.9	Diarrhoea	388	14.3	Hypothyroidism	42	4.7
2	Colitis	65	9.5	Colitis	299	11.1	Diarrhoea	38	4.3
3	Pyrexia	48	7.0	Rash	176	6.5	Pruritus	29	3.3
4	Rash	38	5.6	Fatigue	139	5.1	Alanine aminotransferase increased	21	2.4
5	Hypophysitis	24	3.5	Hypophysitis	125	4.6	Decreased appetite	21	2.4
6	Pneumonitis	21	3.1	Pyrexia	107	3.9	Aspartate aminotransferase increased	20	2.2
7	Hyperthyroidism	20	2.9	Vomiting	99	3.7	Colitis	18	2.0
8	Hypothyroidism	20	2.9	Pruritus	79	2.9	Gamma-glutamyltransferase increased	17	1.9
9	Pruritus	19	2.8	Dehydration	78	2.9	Pneumonitis	16	1.8
10	Hepatitis	18	2.6	Decreased appetite	74	2.7	Hepatic function abnormal	15	1.7
11	Pneumonia	18	2.6	Abdominal pain	67	2.5	Interstitial lung disease	15	1.7
12	General physical health deterioration	17	2.5	Enterocolitis	56	2.1	Leukoderma	15	1.7
13	Abdominal pain	16	2.3	Weight decreased	53	2.0	Blood alkaline phosphatase increased	14	1.6
14	Alanine aminotransferase increased	14	2.1	Anaemia	46	1.7	Acute kidney injury	11	1.2
15	Dehydration	14	2.1	Adrenal insufficiency	42	1.6	Lung disorder	11	1.2
16	Aspartate aminotransferase increased	11	1.6	Sepsis	38	1.4	Sepsis	11	1.2
17	Liver disorder (not further clarified)	11	1.6	Hepatitis	36	1.3	Infusion related reaction	10	1.1
18	Myocarditis	11	1.6	Hyponatraemia	35	1.3	Renal impairment	10	1.1
19	Sepsis	11	1.6	Intestinal perforation	35	1.3	Uveitis	10	1.1
20	Type 1 diabetes mellitus	11	1.6	Hypothyroidism	34	1.3	Adrenal insufficiency|liver disorder|diabetic ketoacidosis	9	1.0
Total	591 reactions	682	-	1130 reactions	2704	-	675 reactions	890	-

## Supplementary Materials

The following are available online at http://www.mdpi.com/2075-4418/8/4/76/s1, Supplement Material S1: Support figures and tables. Supplement Material S2: Reaction statistics and outcome analysis.
