# Supplementary material for: Retrospective Side Effect Profiling of the Metastatic Melanoma Combination Therapy Ipilimumab-Nivolumab Using Adverse Event Data
Source: Diagnostics (Basel). 2018 Oct 31;8(4):76. doi: 10.3390/diagnostics8040076 (PMC6316083; doi:10.3390/diagnostics8040076)
Supplement: Supplementary file 1 [file diagnostics-08-00076-s001.zip › Supplement material S1.pdf]

Table 1: To summarize further the profiles of the three sets (cohorts #6-#8) we complied the union of the top twenty most frequent HLT MedDRA classes and of those that linked with the top twenty most frequent reactions, derived from Table 1 in the main text. Cases that had PRR<=1, p>=0.05, or that occurred in less than 1% of cohort AEs were excluded.

Cohort	Ipilimumab and Nivolumab (#8)			Ipilimumab (#6)			Nivolumab (#7)		
**Reaction names (MedDRA)**	**AEs**	**PRR (95%CI)**	**p<0.05**	**AEs**	**PRR (95%CI)**	**p<0.05**	**AEs**	**PRR (95%CI)**	**p<0.05**
Hypopigmentation disorders	7	51.2 (24.5 - 107.2)	T	11	20.4 (11.3 - 36.8)	T	20	113.1 (73.1 - 174.9)	T
• Leukoderma	4	707.4 (258.6 - 1935.4)	T	-	- -	-	15	2439.2 (1383.4 - 4300.9)	T
• Vitiligo	3	62 (20.0 - 192.3)	T	10	52.7 (28.3 - 98.5)	T	4	63.4 (23.8 - 169.2)	T
Muscle infections and inflammations	5	9.3 (3.9 - 22.3)	T	8	3.8 (1.9 - 7.5)	T	12	17.2 (9.8 - 30.1)	T
Febrile disorders	49	3.2 (2.4 - 4.1)	T	113	1.8 (1.5 - 2.2)	T	21	1 (0.7 - 1.6)	F
• Pyrexia	48	3.6 (2.7 - 4.7)	T	107	2 (1.7 - 2.4)	T	21	1.2 (0.8 - 1.8)	F
Hepatic and hepatobiliary disorders nec	13	4.5 (2.6 - 7.7)	T	20	1.8 (1.1 - 2.7)	T	12	3.2 (1.8 - 5.6)	T
• Liver disorder	11	6.3 (3.5 - 11.3)	T	14	2 (1.2 - 3.4)	T	9	3.9 (2.1 - 7.5)	T
Gastrointestinal and abdominal pains (excl oral and throat)	19	1.1 (0.7 - 1.7)	F	86	1.2 (1.0 - 1.5)	T	12	0.5 (0.3 - 0.9)	T
• Abdominal pain	16	1.8 (1.1 - 2.9)	T	67	1.9 (1.5 - 2.4)	T	10	0.9 (0.5 - 1.6)	F
Noninfectious myocarditis	11	42.3 (23.5 - 76.1)	T	6	5.8 (2.6 - 12.9)	T	6	17.7 (8.0 - 39.2)	T
• Myocarditis	11	47.5 (26.4 - 85.5)	T	6	6.5 (2.9 - 14.5)	T	6	19.8 (8.9 - 44.1)	T
Adrenal cortical hypofunctions	14	32.8 (19.5 - 55.2)	T	47	28 (21.0 - 37.2)	T	13	23.4 (13.6 - 40.1)	T
• Adrenal insufficiency	10	39.6 (21.4 - 73.3)	T	42	42.4 (31.3 - 57.3)	T	9	27.3 (14.2 - 52.3)	T
Renal failure and impairment	18	1.2 (0.8 - 2.0)	F	67	1.2 (0.9 - 1.5)	F	29	1.5 (1.1 - 2.2)	T
• Acute kidney injury	10	4.8 (2.6 - 8.9)	T	17	2.1 (1.3 - 3.3)	T	11	4.1 (2.3 - 7.3)	T
• Renal failure	6	1.2 (0.5 - 2.6)	F	15	0.7 (0.5 - 1.2)	F	8	1.2 (0.6 - 2.4)	F
• Renal failure acute	1	0.3 (0.04 - 1.8)	F	30	2 (1.4 - 2.8)	T	-	- -	-
Pruritus nec	20	1.1 (0.7 - 1.7)	F	109	1.5 (1.3 - 1.8)	T	33	1.4 (1.0 - 2.0)	F
• Pruritus	19	1.8 (1.1 - 2.8)	T	79	1.9 (1.5 - 2.3)	T	29	2.1 (1.5 - 3.0)	T
Diabetic complications nec	8	8.7 (4.4 - 17.3)	T	2	0.5 (0.1 - 2.2)	F	9	7.5 (4.0 - 14.4)	T
• Diabetic ketoacidosis	8	10.5 (5.3 - 20.9)	T	2	0.7 (0.2 - 2.6)	F	9	9 (4.7 - 17.3)	T
Intestinal ulcers and perforation nec	4	3.5 (1.3 - 9.2)	T	72	15.8 (12.6 - 19.8)	T	2	1.3 (0.3 - 5.3)	F
• Intestinal perforation	3	6.7 (2.2 - 20.6)	T	35	19.7 (14.2 - 27.5)	T	1	1.7 (0.2 - 12.1)	F
Appetite disorders	14	1.2 (0.7 - 2.0)	F	80	1.7 (1.4 - 2.1)	T	22	1.4 (0.9 - 2.1)	F
• Decreased appetite	11	1.5 (0.8 - 2.6)	F	74	2.5 (2.0 - 3.1)	T	21	2.2 (1.4 - 3.3)	T
Diabetes mellitus (incl subtypes)	20	3.3 (2.1 - 5.0)	T	12	0.5 (0.3 - 0.9)	T	11	1.4 (0.8 - 2.5)	F
• Type 1 diabetes mellitus	11	52.9 (29.4 - 95.3)	T	2	2.4 (0.6 - 9.7)	F	7	25.8 (12.3 - 54.0)	T
Tissue enzyme analyses nec	5	2.3 (1.0 - 5.5)	F	16	1.8 (1.1 - 3.0)	T	19	6.7 (4.3 - 10.4)	T
Colitis (excl infective)	66	25.4 (20.2 - 31.9)	T	319	31.2 (28.1 - 34.6)	T	20	5.9 (3.8 - 9.1)	T
• Colitis	65	60.9 (48.3 - 76.8)	T	299	72.1 (64.7 - 80.3)	T	18	12.9 (8.2 - 20.4)	T
Infusion site reactions	3	1.2 (0.4 - 3.6)	F	22	2.2 (1.4 - 3.3)	T	11	3.3 (1.8 - 5.9)	T
• Infusion related reaction	3	1.7 (0.6 - 5.3)	F	18	2.6 (1.7 - 4.1)	T	10	4.4 (2.4 - 8.2)	T
Metabolic disorders nec	49	14.7 (11.3 - 19.3)	T	82	6.2 (5.0 - 7.7)	T	57	13.2 (10.2 - 16.9)	T
• Hyperthyroidism	20	43.8 (28.4 - 67.5)	T	16	8.8 (5.4 - 14.4)	T	8	13.4 (6.7 - 26.7)	T
• Hypothyroidism	20	18.4 (11.9 - 28.3)	T	34	7.9 (5.6 - 11.0)	T	42	29.6 (22.0 - 39.8)	T
Diarrhoea (excl infective)	67	3.3 (2.6 - 4.1)	T	399	4.9 (4.5 - 5.4)	T	38	1.4 (1.0 - 1.9)	T
• Diarrhoea	67	3.3 (2.6 - 4.2)	T	388	4.8 (4.4 - 5.3)	T	38	1.4 (1.1 - 2.0)	T
Hypothalamic and pituitary disorders nec	27	50.4 (34.8 - 73.0)	T	150	72 (61.5 - 84.3)	T	10	14.3 (7.7 - 26.4)	T
• Hypophysitis	24	621.1 (414.8 - 930.0)	T	125	1051.1 (860.0 - 1284.8)	T	6	114.4 (51.3 - 255.3)	T
Arthropathies nec	13	2.7 (1.6 - 4.6)	T	9	0.5 (0.2 - 0.9)	T	7	1.1 (0.5 - 2.3)	F
• Arthritis	9	3.3 (1.7 - 6.3)	T	7	0.7 (0.3 - 1.4)	F	4	1.1 (0.4 - 3.0)	F
Pituitary analyses anterior	9	11.6 (6.1 - 22.3)	T	21	6.9 (4.5 - 10.5)	T	12	12 (6.8 - 20.9)	T
Gastrointestinal inflammatory disorders nec	15	4.8 (2.9 - 7.9)	T	69	5.5 (4.4 - 7.0)	T	10	2.4 (1.3 - 4.5)	T
• Enterocolitis	6	37.9 (17.1 - 84.1)	T	56	91.6 (70.4 - 119.2)	T	6	29 (13.1 - 64.5)	T
Lower respiratory tract inflammatory and immunologic conditions	22	9.7 (6.4 - 14.6)	T	38	4.2 (3.1 - 5.8)	T	21	7.1 (4.6 - 10.8)	T
• Pneumonitis	21	27.9 (18.3 - 42.5)	T	33	11.1 (7.9 - 15.6)	T	16	16.3 (10.0 - 26.5)	T
Respiratory tract disorders nec	9	2 (1.0 - 3.8)	F	9	0.5 (0.3 - 1.0)	T	13	2.2 (1.3 - 3.8)	T
• Lung disorder	7	4.1 (2.0 - 8.6)	T	6	0.9 (0.4 - 2.0)	F	11	4.9 (2.7 - 8.9)	T
Liver function analyses	29	3.2 (2.2 - 4.5)	T	80	2.2 (1.8 - 2.7)	T	46	3.8 (2.9 - 5.1)	T
Rashes, eruptions and exanthems nec	49	2.6 (2.0 - 3.4)	T	208	2.8 (2.5 - 3.2)	T	29	1.2 (0.8 - 1.7)	F
• Rash	38	2.8 (2.1 - 3.8)	T	176	3.3 (2.9 - 3.8)	T	26	1.5 (1.0 - 2.2)	F
Hepatic enzymes and function abnormalities	9	4.6 (2.4 - 8.7)	T	17	2.2 (1.4 - 3.5)	T	16	6.2 (3.8 - 10.1)	T
• Hepatic function abnormal	8	5.9 (2.9 - 11.7)	T	7	1.3 (0.6 - 2.7)	F	15	8.5 (5.1 - 14.0)	T

Thyroid analyses	10	29.2 (15.8 - 54.0)	T	6	4.4 (2.0 - 9.8)	T	13	29.1 (17.0 - 50.0)	T
Anaemias nec	5	0.6 (0.2 - 1.4)	F	48	1.4 (1.1 - 1.8)	T	12	1.1 (0.6 - 1.9)	F
• Anaemia	5	0.6 (0.3 - 1.5)	F	46	1.5 (1.1 - 2.0)	T	12	1.2 (0.7 - 2.1)	F
Sepsis, bacteraemia, viraemia and fungaemia nec	14	2 (1.2 - 3.4)	T	50	1.8 (1.4 - 2.4)	T	18	2 (1.3 - 3.1)	T
• Sepsis	11	2.5 (1.4 - 4.6)	T	38	2.2 (1.6 - 3.1)	T	11	2 (1.1 - 3.5)	T
Iris and uveal tract infections, irritations and inflammations	5	10.5 (4.4 - 25.2)	T	21	11.2 (7.3 - 17.2)	T	11	17.8 (9.9 - 32.0)	T
• Uveitis	5	17.3 (7.2 - 41.5)	T	14	12.3 (7.3 - 20.7)	T	10	26.6 (14.3 - 49.3)	T
Total fluid volume decreased	14	2.2 (1.3 - 3.8)	T	78	3.2 (2.5 - 4.0)	T	6	0.7 (0.3 - 1.6)	F
• Dehydration	14	2.4 (1.5 - 4.1)	T	78	3.4 (2.8 - 4.3)	T	6	0.8 (0.4 - 1.8)	F
Hepatocellular damage and hepatitis nec	41	9.7 (7.2 - 13.0)	T	82	4.9 (4.0 - 6.1)	T	20	3.6 (2.4 - 5.6)	T
• Autoimmune hepatitis	9	39.9 (20.8 - 76.3)	T	28	31.5 (21.7 - 45.6)	T	6	20.3 (9.2 - 45.2)	T
• Hepatitis	18	17.9 (11.3 - 28.2)	T	36	9 (6.5 - 12.5)	T	7	5.3 (2.5 - 11.1)	T
• Hepatotoxicity	7	10.9 (5.2 - 22.8)	T	8	3.2 (1.6 - 6.3)	T	3	3.6 (1.2 - 11.1)	F
Sodium imbalance	9	3.7 (1.9 - 7.1)	T	35	3.6 (2.6 - 5.1)	T	8	2.5 (1.3 - 5.0)	T
• Hyponatraemia	8	3.6 (1.8 - 7.3)	T	35	4 (3.0 - 5.6)	T	7	2.4 (1.2 - 5.1)	T

Table 2: Top twenty reported reactions in cohorts #3-#5 based on frequency (upper part of the table) and PRR disproportionality score (lower part of the table). Reaction terms highlighted in **bold** were part of both lists. We considered only statistically significant (at 0.05) reactions with PRR >1 that occurred in more that 1% of each cohort's AEs, and excluded the following terms: 'death', 'inappropriate schedule of drug administration', 'infusion related reaction', 'malignant neoplasm progression', 'metastases to central nervous system', 'neoplasm malignant', 'off label use', 'prescribed overdose', 'transfusion'.

Cohort:	#3: Ipilimumab and Nivolumab (together)				#4: Ipilimumab w/o Nivolumab				#5: Nivolumab w/o Ipilimumab			
Top 20 reactions	NAME	AEs	PRR	%	NAME	AEs	PRR	%	NAME	AEs	PRR	%
By Frequency	DIARRHOEA	175	3.8	11.1	**DIARRHOEA**	759	5.3	15.7	DIARRHOEA	66	1.6	4.8
	**COLITIS**	164	67.2	10.4	**COLITIS**	577	79.5	11.9	**HYPOTHYROIDISM**	65	29.8	4.7
	PYREXIA	139	4.5	8.8	RASH	282	2.9	5.8	PYREXIA	60	2.2	4.4
	RASH	60	1.9	3.8	FATIGUE	279	1.6	5.8	**ALANINE AMINOTRANSFERASE INCREASED**	56	9.4	4.1
	**HYPOPHYSITIS**	58	703.3	3.7	PYREXIA	272	2.9	5.6	**ASPARTATE AMINOTRANSFERASE INCREASED**	51	9.5	3.7
	DEHYDRATION	55	4.2	3.5	NAUSEA	243	1.2	5.0	**HEPATIC FUNCTION ABNORMAL**	41	15.0	2.9
	VOMITING	54	1.3	3.4	**HYPOPHYSITIS**	222	1442.7	4.6	RASH	41	1.5	2.9
	**PNEUMONITIS**	52	30.0	3.3	VOMITING	219	1.7	4.5	PRURITUS	40	1.9	2.9
	**Acute kidney injury**	49	10.3	3.1	**DEHYDRATION**	216	5.3	4.5	DECREASED APPETITE	38	2.5	2.8
	**ADRENAL INSUFFICIENCY**	45	78.1	2.9	ABDOMINAL PAIN	147	2.3	3.0	**INTERSTITIAL LUNG DISEASE**	33	10.3	2.4
	**HYPERTHYROIDISM**	45	42.9	2.9	PRURITUS	142	1.9	2.9	**GAMMA-GLUTAMYLTRANSFERASE INCREASED**	32	13.8	2.3
	**HYPOTHYROIDISM**	41	16.3	2.6	DECREASED APPETITE	139	2.6	2.9	**COLITIS**	31	14.4	2.3
	ALANINE AMINOTRANSFERASE INCREASED	40	5.8	2.5	ANAEMIA	113	2.0	2.3	**BLOOD ALKALINE PHOSPHATASE INCREASED**	30	10.8	2.2
	**HEPATITIS**	37	15.9	2.4	**HYPONATRAEMIA**	107	6.9	2.2	ANAEMIA	28	1.8	2.0
	ABDOMINAL PAIN	34	1.6	2.2	**ADRENAL INSUFFICIENCY**	105	60.5	2.2	**LEUKODERMA**	26	3435.6	1.9
	**AUTOIMMUNE HEPATITIS**	34	65.9	2.2	**SEPSIS**	102	3.3	2.1	**PNEUMONITIS**	25	16.6	1.8
	GENERAL PHYSICAL HEALTH DETERIORATION	34	4.1	2.2	WEIGHT DECREASED	98	1.4	2.0	GENERAL PHYSICAL HEALTH DETERIORATION	21	2.9	1.5
	ASPARTATE AMINOTRANSFERASE INCREASED	32	5.2	2.0	HYPOTENSION	92	1.7	1.9	**AUTOIMMUNE HEPATITIS**	20	44.3	1.5
	SEPSIS	31	3.1	1.9	**ALANINE AMINOTRANSFERASE INCREASED**	85	4.0	1.8	**Acute kidney injury**	20	4.8	1.5
	HYPONATRAEMIA | HYPOTENSION	30	5.9 | 1.7	1.9	**ASPARTATE AMINOTRANSFERASE INCREASED**	85	4.5	1.8	**RENAL FAILURE**	20	1.9	1.5
By PRR	NAME	AEs	PRR	%	NAME	AEs	PRR	%	NAME	AEs	PRR	%
	Autoimmune colitis	22	1463.8	1.4	**HYPOPHYSITIS**	222	1442.7	4.6	**LEUKODERMA**	26	3435.6	1.9
	**HYPOPHYSITIS**	58	703.3	3.7	HYPOPITUITARISM	57	231.9	1.2	**AUTOIMMUNE HEPATITIS**	20	44.3	1.5
	HYPOPITUITARISM	22	253.4	1.4	**COLITIS**	577	79.5	11.9	**HYPOTHYROIDISM**	65	29.8	4.8
	THYROIDITIS	17	105.9	1.1	ENTEROCOLITIS	81	75.1	1.7	ADRENAL INSUFFICIENCY	15	29.6	1.1
	**ADRENAL INSUFFICIENCY**	45	78.1	2.9	**ADRENAL INSUFFICIENCY**	105	60.5	2.2	UVEITIS	16	27.7	1.2
	**COLITIS**	164	67.2	10.4	LARGE INTESTINE PERFORATION	66	34.0	1.4	BLOOD THYROID STIMULATING HORMONE INCREASED	15	27.5	1.1
	**AUTOIMMUNE HEPATITIS**	34	65.8	2.2	LIPASE INCREASED	62	25.1	1.3	MYOSITIS	14	21.8	1.1
	ENTEROCOLITIS	17	46.8	1.01	INTESTINAL PERFORATION	75	23.8	1.6	**PNEUMONITIS**	25	16.6	1.8
	**HYPERTHYROIDISM**	45	42.9	2.9	PNEUMONITIS	60	11.3	1.2	HYPERTHYROIDISM	15	16.3	1.1
	TYPE 1 DIABETES MELLITUS	17	35.5	1.1	RASH MACULO-PAPULAR	51	9.6	1.1	**HEPATIC FUNCTION ABNORMAL**	41	15.0	2.9
	MYOCARDITIS	18	33.8	1.1	HYPOTHYROIDISM	70	9.1	1.5	**COLITIS**	31	14.4	2.3
	**PNEUMONITIS**	52	30.0	3.3	HEPATITIS	59	8.3	1.2	**GAMMA-GLUTAMYLTRANSFERASE INCREASED**	32	13.8	2.3
	LIPASE INCREASED	17	20.9	1.1	**HYPONATRAEMIA**	107	6.9	2.2	**BLOOD ALKALINE PHOSPHATASE INCREASED**	30	10.8	2.2
	**HYPOTHYROIDISM**	41	16.3	2.6	**DEHYDRATION**	216	5.3	4.5	DIABETIC KETOACIDOSIS	16	10.4	1.2
	**HEPATITIS**	37	15.9	2.4	**DIARRHOEA**	759	5.3	15.7	**INTERSTITIAL LUNG DISEASE**	33	10.3	2.4
	HEPATOTOXICITY	18	12.2	1.1	**ASPARTATE AMINOTRANSFERASE INCREASED**	85	4.5	1.8	**ASPARTATE AMINOTRANSFERASE INCREASED**	51	9.5	3.7
	TRANSAMINASES INCREASED	20	10.9	1.3	HYPOKALAEMIA	50	4.2	1.0	**ALANINE AMINOTRANSFERASE INCREASED**	56	9.4	4.1
	**Acute kidney injury**	49	10.3	3.1	**ALANINE AMINOTRANSFERASE INCREASED**	85	4.0	1.8	C-REACTIVE PROTEIN INCREASED	15	6.5	1.1
	DIABETIC KETOACIDOSIS	17	9.6	1.1	Acute kidney injury	49	3.3	1.0	**Acute kidney injury**	20	4.8	1.5
	LIVER DISORDER	28	6.9	1.8	**SEPSIS**	102	3.3	2.1	LUNG DISORDER	15	4.4	1.1

Figure 1: Simplified overview of the mechanisms of action of checkpoint inhibitors. PD-1 antibodies (nivolumab, pembrolizumab) prevent the interaction of PD-1 with its ligand PD-L1. CTLA-4 antibodies (ipilimumab, tremelimumab) block the interaction between CTLA-4 and its receptor CD80. Both types of inhibitors lead to the activation of the T-cell. Checkpoint inhibitors play also key role in autoimmune conditions. One such example is myocarditis that patients may develop via T-cell infiltration at the site of myocardium [1]. Such massive tissue destruction was observed also in genetically modified mouse models with impaired CTLA4 expression [2]. Second, tumor neoantigens and antigens from healthy tissue could cross-react. Development of vitiligo and other hypopigmentation diseases in melanoma patients under immunotherapy illustrates this situation where normal melanocytes are also being targeted by immune cells [3]. In addition to increased T-cell activity, expansion of already present autoantibodies could account for adverse effects, like thyroiditis [4]. Moreover, secretion of inflammatory cytokines in response to treatment has been observed in the case of colitis both in patients and animal models [5]. Finally, normal tissues actually expressing the checkpoint molecules (i.e. CTLA4 in the pituitary gland) may exhibit inflammation enhancement via activation of the complement.

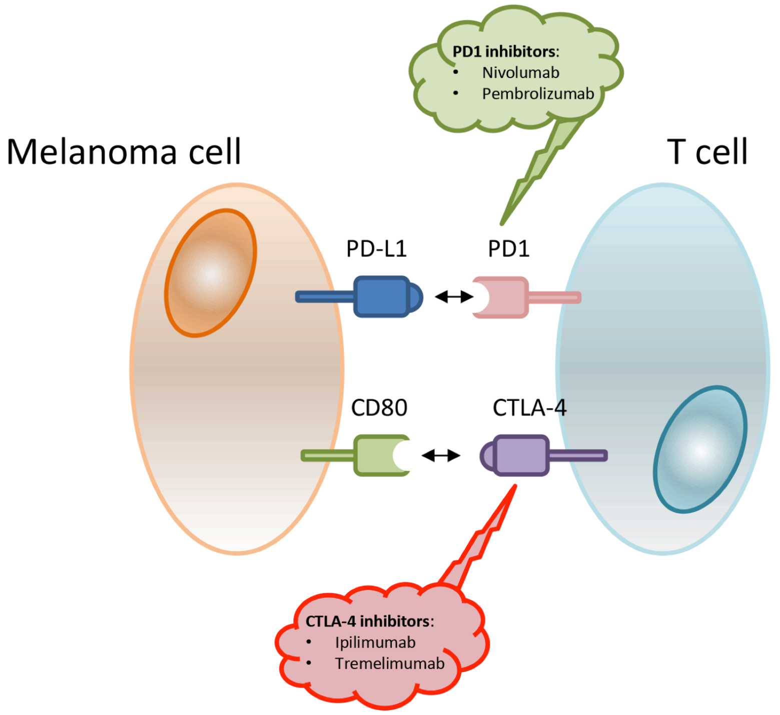

Figure 2: Distribution of outcomes 'Death' and 'Hospitalization' in each cohort. We contrasted the reporting of 'Death' and 'Hospitalization' as outcomes between the examined cohorts #1-#8 and (a) the overall FAERS dataset (named 'FAERS'), AE cases where indication term(s) linked to the highest level MedDRA term 'Neoplasms benign, malignant and unspecified (incl cysts and polyps)' (named 'Cancer'), and the 'general melanoma' patient (named 'Skin melanomas'; defined as the AE cases with indication(s) linked to the HLT MedDRA term 'Skin melanomas (excl ocular)'). In comparison to the 'general melanoma patient' in FAERS, we observe reduced death occurrence, especially for the ipilimumab and the ipilimumab plus nivolumab groups. Also, note that direct comparison to the 'general melanoma patient' is somewhat unfair without an exact disease stage dissemination of those patients. We also compared the occurrence of these outcomes in the presence and absence of reactions from each cohort's side effect profile: in (b) we statistically measure the difference in cohorts #3-#8, when all reactions from each side-effect profile are considered together for the respective cohort – in all cases, 'Death' is under-represented (PRR <1) and 'Hospitalization' is over-represented (PRR >1) upon manifestation of the respective reactions; all associations are statistically significant (*p*-value<0.05) except for 'Death' in cohort #5.

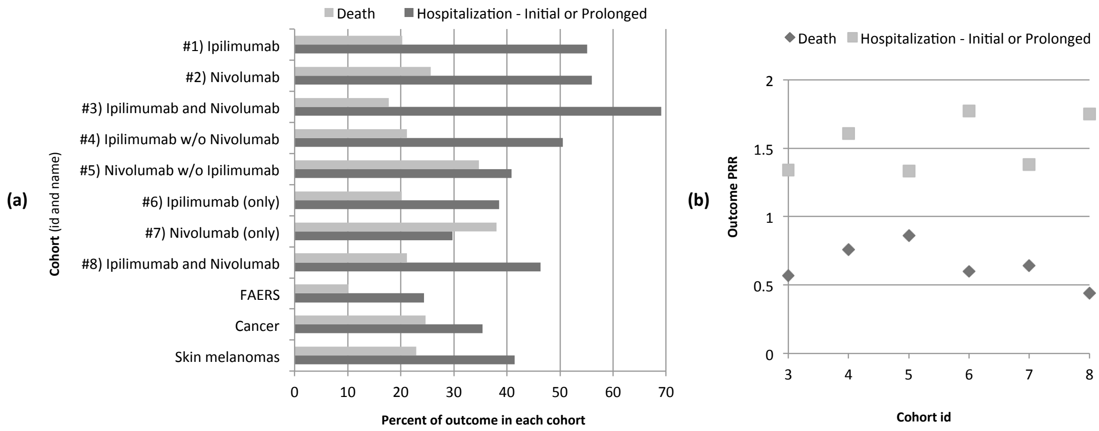

Figure 3: Radiologic manifestations of irAEs. Despite sometimes being clinically silent, irAEs are very often associated with radiologic manifestations. Towards successful management, radiologists should be educated on irAE imaging patterns, since early detection can lead to timely discontinuation of immunotherapy and the initiation of corticosteroid treatment. To this end, morphologic techniques like computed tomography (CT) and magnetic resonance imaging (MRI) can be used, together with more novel, functional imaging modalities, like positron emission tomography/computed tomography (PET/CT) that provides metabolic tissue assessment. This is of particular significance, since functional/metabolic tissue changes most of the times precede respective anatomic changes [6]. The illustration presents one such PET/CT example with the radiotracer fluorodeoxyglucose (^{18}F-FDG) of an of an advanced melanoma patient demonstrating radiologic signs of colitis undergoing a 4-cycle ipilimumab treatment. (A) Baseline PET/CT before onset of treatment demonstrated metastatic lesions in the left lung and the right leg. (B) ^{18}F-FDG PET/CT performed after two cycles of ipilimumab revealed progressive disease with increase in the number, size and metabolism of the metastatic lesions and at the same time diffusely increased tracer uptake in the descending colon, indicative of colitis. (C) PET/CT performed after the end of the four-cycle ipilimumab treatment demonstrated progressive disease with further increase in the number and intensity of uptake of the metastatic lesions, as well as persistence and expansion of the immune-related colitis in the transverse and upper ascending colon. (D) One month after the end of immunotherapy, PET/CT revealed dramatic progression of the metastatic disease and remission of the colitis.

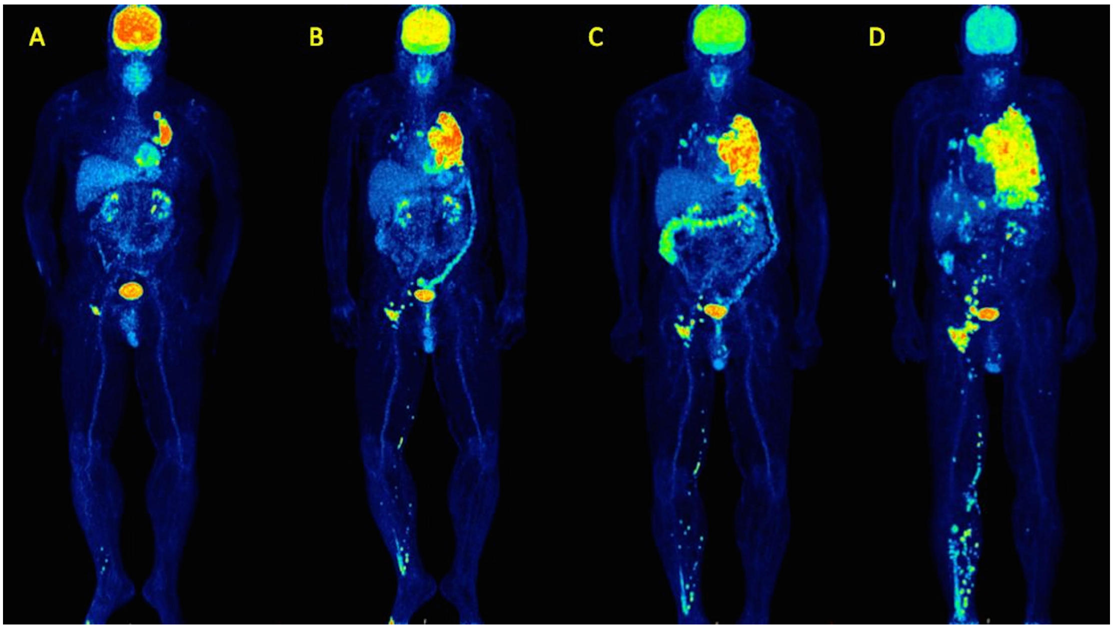

References:

1. Johnson, D. B. *et al.* Fulminant Myocarditis with Combination Immune Checkpoint Blockade. *N. Engl. J. Med.* **375**, 1749–1755 (2016).
2. Tivol, E. A. *et al.* Loss of CTLA-4 leads to massive lymphoproliferation and fatal multiorgan tissue destruction, revealing a critical negative regulatory role of CTLA-4. *Immunity* **3**, 541–547 (1995).
3. Byrne, E. H. & Fisher, D. E. Immune and molecular correlates in melanoma treated with immune checkpoint blockade. *Cancer* **123**, 2143–2153 (2017).
4. Osorio, J. C. *et al.* Antibody-mediated thyroid dysfunction during T-cell checkpoint blockade in patients with non-small-cell lung cancer. *Ann. Oncol.* **28**, 583–589 (2017).
5. Callahan, M. K. *et al.* Evaluation of serum IL-17 levels during ipilimumab therapy: Correlation with colitis. *JCO* **29**, 2505–2505 (2011).
6. Wahl, R. L., Jacene, H., Kasamon, Y. & Lodge, M. A. From RECIST to PERCIST: Evolving Considerations for PET response criteria in solid tumors. *J. Nucl. Med.* **50 Suppl 1**, 122S–50S (2009).
